# CD44 Gene Polymorphisms and Environmental Factors on Oral Cancer Susceptibility in Taiwan

**DOI:** 10.1371/journal.pone.0093692

**Published:** 2014-04-03

**Authors:** Ying-Erh Chou, Ming-Ju Hsieh, Chung-Han Hsin, Whei-Ling Chiang, Yi-Cheng Lai, Yu-Hsien Lee, Shu-Ching Huang, Shun-Fa Yang, Chiao-Wen Lin

**Affiliations:** 1 Institute of Medicine, Chung Shan Medical University, Taichung, Taiwan; 2 Cancer Research Center, Changhua Christian Hospital, Changhua, Taiwan; 3 School of Medicine, Chung Shan Medical University, Taichung, Taiwan; 4 Department of Otolaryngology, Chung Shan Medical University Hospital, Taichung, Taiwan; 5 School of Medical Laboratory and Biotechnology, Chung Shan Medical University, Taichung, Taiwan; 6 School of Dentistry, Chung Shan Medical University, Taichung, Taiwan; 7 Department of Dentistry, Chung Shan Medical University Hospital, Taichung, Taiwan; 8 Department of Medical Research, Chung Shan Medical University Hospital, Taichung, Taiwan; 9 Institute of Oral Sciences, Chung Shan Medical University, Taichung, Taiwan; University of Chicago, Department of Medicine, United States of America

## Abstract

**Background:**

Oral squamous cell carcinoma (OSCC) is the fourth leading cause of male cancer death in Taiwan. Exposure to environmental carcinogens is the primary risk factor for developing OSCC. CD44, a well-known tumor marker, plays a crucial role in tumor cell differentiation, invasion, and metastasis. This study investigated CD44 single-nucleotide polymorphisms (SNPs) with environmental risk factors to determine OSCC susceptibility and clinicopathological characteristics.

**Methodology/Principal Findings:**

Real-time polymerase chain reaction (PCR) was used to analyze 6 SNPs of CD44 in 599 patients with oral cancer and 561 cancer-free controls. We determined that the CD44 rs187115 polymorphism carriers with the genotype AG, GG, or AG+GG were associated with oral cancer susceptibility. Among 731 smokers, CD44 polymorphisms carriers with the betel-nut chewing habit had a 10.30–37.63-fold greater risk of having oral cancer compared to CD44 wild-type (WT) carriers without the betel-nut chewing habit. Among 552 betel-nut chewers, CD44 polymorphisms carriers who smoked had a 4.23–16.11-fold greater risk of having oral cancer compared to those who carried the WT but did not smoke. Finally, we also observed that the stage III and IV oral cancer patients had higher frequencies of CD44 rs187115 polymorphisms with the variant genotype (AG+GG) compared with the wild-type (WT) carriers.

**Conclusion:**

Our results suggest that gene–environment interactions between the CD44 polymorphisms and betel quid chewing and tobacco smoking increase the susceptibility to oral cancer development. Patients with CD44 rs187115 variant genotypes (AG+GG) were correlated with a higher risk of oral cancer development, and these patients may possess greater chemoresistance to advanced- to late-stage oral cancer than WT carriers do. The CD44 rs187115 polymorphism has potential predictive significance in oral carcinogenesis and also may be applied as factors to predict the clinical stage in OSCC patients.

## Introduction

Oral squamous cell carcinoma (OSCC) is a common malignant cancer in the head and neck region. It is not only the sixth most common cancer worldwide but also the fourth leading cause of male cancer death in Taiwan [1], [2]. Despite evolving imaging techniques providing more precise detection and staging, combined with advances in surgery, chemotherapy, and radiation, the prognosis and mortality of OSCC has remained stable [3], [4]. Carcinogen exposure is the primary risk factor for developing OSCC; specifically, previous studies have indicated that betel-quid chewing, tobacco smoking, and alcohol consumption are the primary risk factors associated with OSCC development [5]–[7].

CD44 glycoproteins are members of the hyaluronate receptor and are associated with numerous fundamental biological processes, such as lymphocyte homing, cell migration, inflammation, hematopoiesis, wound healing, apoptosis, and embryonal development [8]. Despite its regulation in many cellular processes, CD44 plays a crucial role in tumor cell differentiation, invasion, and metastasis [9], [10]. CD44^+^ cells are proposed to be cancer stem cells (CSCs) because CD44 is a well-known marker of breast-cancer-initiating cells (BCICs) [10], [11]. CD44^+^ cells in mice engraft at higher frequencies and have enhanced chemoresistance [11]–[13]. Furthermore, CD44^+^ cells are also involved in the epithelial to mesenchymal transition (EMT), which is a genetic program associated with metastasis [14]. Although regulation of CD44 expression in head and neck cancers remains incompletely understood, recent studies have demonstrated and suggested that the increased CD44 expression in OSCC is correlated with increased metastasis, recurrence, resistance to chemo- and radiation therapy, and decreased survival [13], [15]–[17].

Single nucleotide polymorphisms (SNPs) are the most common type of DNA sequence variation, and the expression of certain genes may be affected by their genetic variations [18], [19]. Previous studies have documented the impact of CD44 polymorphisms on human cancer susceptibility [20]–[24]. CD44 genetic variants have been identified as playing a substantial regulatory role in cellular stress responses to chemotherapeutic agents and further affecting sarcoma incidence and survival [20]. The germline polymorphisms in colon CSC genes have demonstrated that CD44 is involved in predicting tumor recurrence in patients with colorectal cancer [21]. In breast cancer, the SNPs of CD44 are suggested to affect breast cancer development and prognosis by increasing CD44 expression [22], [23]. Furthermore, CD44 polymorphisms, alone or in combination, may act as markers for identifying localized gastric adenocarcinoma patients at a high risk of tumor recurrence [24]. Thus, we hypothesized that CD44 polymorphisms play a critical role in oral cancer development.

The effects of CD44 on human cancer metastasis and prognosis have been well documented, but the effects of CD44 gene SNPs and environmental carcinogens on oral cancer susceptibility and clinical features remain poorly investigated. In the present study, a case–control investigation was performed for 6 SNPs located in the promoter region or the 3′UTR of CD44 to analyze their contribution and the associations between environmental factors and oral cancer clinicopathologic characteristics.

## Materials and Methods

### Subjects and Specimen Collection

In 2007–2012, we recruited 599 patients (577 males and 22 females with a mean age of 54.34±11.28 years) at Chung Shan Medical University Hospital in Taichung and Changhua Christian Hospital and Show Chwan Memorial Hospital in Changhua, Taiwan as the case group. For the control group, we randomly chose 561 non-cancer individuals (457 males and 104 females with a mean age of 51.81±14.71 years) who visited those same hospitals and thus were from the same geographic area. For both cases and controls, we used a questionnaire to obtain exposure information about betel-quid chewing, tobacco use, and alcohol consumption. Medical information of the cases, including TNM clinical staging, the primary tumor size, lymph node involvement, and histologic grade, was obtained from their medical records. Oral-cancer patients were clinically staged at the time of their diagnosis according to the TNM staging system of the American Joint Committee on Cancer (AJCC) Staging Manual (7th ed.). Stage I = T1N0M0; Stage II = T2N0M0; Stage III = T3N0M0, or T1, T2 or T3N1M0; Stage IV = any T4 lesion, any N2 or N3 lesion, or any M1 lesion. Tumor differentiation was examined by a pathologist according to the AJCC classification. Whole-blood specimens collected from controls and OSCC patients were placed in tubes containing ethylenediaminetetraacetic acid (EDTA), were immediately centrifuged, and then stored at −80°C. This study was approved by the Institutional Review Boards of Show Chwan Memorial Hospital, and informed written consent to participate in the study was obtained from each individual.

### Selection of CD44 Polymorphisms

A total of six SNPs in CD44 were selected from the International HapMap Project data for this study. We included the SNP rs1425802 in the promoter region. Three SNPs (rs11821102, rs10836347 and rs13347) which locate in the 3′UTR of CD44 were selected in this study since these SNPs were found to affect binding ability of certain MicroRNA in a Chinese population [23]. Furthermore, the other SNPs (rs187115 and rs713330) were selected in this study because the gene polymorphisms of these SNPs have been found to associate with gastric and breast cancers [23], [24].

### Genomic DNA Extraction

Genomic DNA was extracted using QIAamp DNA blood mini kits (Qiagen, Valencia, CA, USA) following the manufacturer’s instructions. We dissolved DNA in TE buffer (10 mM Tris and 1 mM EDTA; pH 7.8) and then quantified it by measuring the optical density at 260 nm. The final preparation was stored at −20°C and used to create templates for the polymerase chain reaction (PCR) [25].

### Real-time PCR

Allelic discrimination of the rs1425802, rs187115, rs713330, rs11821102, rs10836347 and rs13347 polymorphisms of the CD44 gene was assessed with the ABI StepOne Real-Time PCR System (Applied Biosystems, Foster City, CA, USA), and analyzed with SDS vers. 3.0 software (Applied Biosystems) using the TaqMan assay. The final volume for each reaction was 5 μL, containing 2.5 μL TaqMan Genotyping Master Mix, 0.125 μL TaqMan probe mix, and 10 ng genomic DNA. The real-time PCR included an initial denaturation step at 95°C for 10 min, followed by 40 cycles at of 95°C for 15 s and then at 60°C for 1 min [26], [27].

### Statistical Analysis

Differences between the 2 groups were considered significant if *p* values were <0.05. Hardy-Weinberg equilibrium (HWE) was assessed using a goodness-of-fit *Χ^2^*-test for biallelic markers. The Mann-Whitney *U*-test and Fisher’s exact test were used to compare differences in the distributions of patient demographic characteristics between the non-cancer (control) and oral-cancer groups. The adjusted odds ratios (ORs) and 95% confidence intervals (CIs) of the association between genotype frequencies and risk plus clinicopathological characteristics were estimated using multiple logistic regression models, after controlling for other covariates. We analyzed all data with Statistical Analytic System (SAS Institute, Cary, NC, USA) for Windows.

## Results

The statistical analysis of demographical characteristics is shown in Table 1. We found significantly different distributions of age (control: 51.81±14.71; oral cancer: 54.34±11.28; *p* = 0.001) and gender (control: 457 males and 104 females; oral cancer: 577 males and 22 females; *p*<0.0001) between the two groups. Significant differences were observed in the distributions of betel-quid chewing (*p*<0.0001), alcohol consumption (*p*<0.0001), and tobacco smoking (*p*<0.0001) between controls and OSCC patients.

**Table 1:** The distributions of demographical characteristics in 561 controls and 599 patients with oral cancer.

Variable	Controls (N = 561)	Patients (N = 599)	*p* value
**Age (yrs)**	**Mean ± S.D.**	**Mean ± S.D.**	
	51.81±14.71	54.34±11.28	*p* = 0.001*
**Gender**	**n (%)**	**n (%)**	
Male	457 (81.5%)	577 (96.3%)	
Female	104 (18.5%)	22 (3.7%)	*p*<0.0001*
**Betel nut chewing**			
No	468 (83.4%)	140 (23.4%)	
Yes	93 (16.6%)	459 (76.6%)	*p*<0.0001*
**Alcohol consumption**			
No	347 (61.9%)	243 (40.6%)	
Yes	214 (38.1%)	356 (59.4%)	*p*<0.0001*
**Tobacco consumption**			
No	341 (60.8%)	88 (14.7%)	
Yes	220 (39.2%)	511 (85.3%)	*p*<0.0001*

Mann-Whitney U test or Fisher’s exact test was used between healthy controls and patients with oral cancer.

*p value <0.05 as statistically significant.

Genotype distributions and associations between oral cancer and CD44 gene polymorphisms are shown in Table 2. Alleles with the highest distribution frequency for the rs1425802, rs187115, rs713330, rs11821102, rs10836347, and rs13347 genes of CD44 in both the healthy control and oral cancer patients respectively were heterozygous for A/G, homozygous for AA, homozygous for T/T, homozygous for G/G, homozygous for C/C, and homozygous for C/C. After adjusting for several variables, no significant differences were observed in the OSCC patients with rs1425802, rs713330, rs11821102, rs10836347, and rs13347 polymorphisms of the CD44 gene compared with those with the wild type (WT). However, patients with the CD44 polymorphic rs187115 AG and GG genotypes exhibited a significantly (*p*<0.05) higher risk of 2.098–(95% confidence interval (CI) = 1.448–3.039) and 2.988–(95% CI = 1.280–6.973), of having OSCC compared with the corresponding WT homozygous patients. Moreover, a similar result was also observed in patients with the CD44 polymorphic rs187115 AG+GG genotypes. Furthermore, in the males group, patients with the CD44 polymorphic rs187115 AG and GG genotypes exhibited a significantly (*p*<0.01) higher risk of 1.810–(95% confidence interval (CI) = 1.217–2.693) and 3.215–(95% CI = 1.348–7.664), of having OSCC compared with the corresponding WT homozygous patients.

**Table 2:** Distribution frequency of *CD44* genotypes in 561 healthy controls and 599 oral cancer patients.

Variable	Controls (N = 561) n (%)	Patients (N = 595) n (%)	OR (95% CI)	AOR (95% CI)
**rs1425802**				
AA	194 (34.6%)	197 (32.9%)	1.00	1.00
AG	235 (41.9%)	249 (41.6%)	1.043 (0.799–1.362)	1.150 (0.784–1.686)
GG	132 (23.5%)	153 (25.5%)	1.141 (0.841–1.550)	1.359 (0.871–2.120)
AG+GG	367 (65.4%)	402 (67.1%)	1.079 (0.846–1.376)	1.222 (0.861–1.732)
**rs187115**				
AA	403 (71.8%)	336 (56.1%)	1.00	1.00
AG	143 (25.5%)	227 (37.9%)	**1.904 (1.476–2.456)** *	**2.098 (1.448–3.039)** *
GG	15 (2.7%)	36 (6.0%)	**2.879 (1.549–5.348)** *	**2.988 (1.280–6.973)** *
AG+GG	158 (28.2%)	263 (43.9%)	**1.996 (1.563–2.550)** *	**2.191 (1.535–3.126)** *
**rs713330**				
TT	467 (83.2%)	507 (84.6%)	1.00	1.00
TC	86 (15.4%)	88 (14.7%)	0.943 (0.683–1.301)	0.950 (0.597–1.510)
CC	8 (1.4%)	4 (0.7%)	0.461 (0.138–1.540)	0.694 (0.102–4.702)
TC+CC	94 (16.8%)	92 (15.4%)	0.902 (0.659–1.234)	0.936 (0.594–1.476)
**rs11821102**				
GG	481 (85.7%)	531 (88.6%)	1.00	1.00
GA	75 (13.4%)	63 (10.5%)	0.761 (0.532–1.087)	0.727 (0.437–1.210)
AA	5 (0.9%)	5 (0.8%)	0.906 (0.261–3.148)	1.227 (0.235–6.394)
GA+AA	80 (14.3%)	68 (11.4%)	0.770 (0.545–1.088)	0.756 (0.462–1.237)
**rs10836347**				
CC	487 (86.8%)	522 (87.1%)	1.00	1.00
CT	69 (12.3%)	73 (12.2%)	0.987 (0.695–1.403)	0.975 (0.584–1.626)
TT	5 (0.9%)	4 (0.7%)	0.746 (0.199–2.796)	0.601 (0.071–5.077)
CT+TT	74 (13.2%)	77 (12.9%)	0.971 (0.690–1.367)	0.953 (0.577–1.575)
**rs13347**				
CC	295 (52.6%)	287 (47.9%)	1.00	1.00
CT	223 (39.8%)	262 (43.8%)	1.208 (0.949–1.537)	1.334 (0.936–1.901)
TT	43 (7.6%)	50 (8.3%)	1.195 (0.771–1.854)	1.069 (0.555–2.058)
CT+TT	266 (47.4%)	312 (52.1%)	1.206 (0.957–1.518)	1.290 (0.918–1.811)

The odds ratios (ORs) and with their 95% confidence intervals (CIs) were estimated by logistic regression models. The adjusted odds ratios (AORs) with their 95% confidence intervals (CIs) were estimated by multiple logistic regression models after controlling for age, gender, betel nut chewing, tobacco and alcohol consumption.

*p value <0.05 as statistically significant.

Interactive effects between environmental risk factors and genetic polymorphisms of CD44 are shown in Tables 3 and 4. Among 731 smokers, subjects with either at least 1 G allele of rs1425802 or rs187115, 1 C allele of rs713330, 1 A allele of rs11821102, 1 T allele of rs10836347 or rs13347, or the betel-nut-chewing habit respectively had 5.443–(95% CI: 2.919–10.148), 10.519–(95% CI: 6.15–17.979), 11.144–(95% CI: 6.923–17.937), 13.055–(95% CI: 7.984–21.348), 13.455–(95% CI: 8.292–21.832), and 5.703-fold (95% CI: 3.203–10.156) higher risks of having oral cancer. Individuals with at least 1 G allele of rs1425802 or rs187115, 1 C allele of rs713330, 1 A allele of rs11821102, 1 T allele of rs10836347 or rs13347, and who chewed betel nut had respective 21.205–(95% CI: 10.901–41.248), 37.631–(95% CI: 18.366–77.106), 23.333–(95% CI: 9.461–57.544), 10.295–(95% CI: 4.400–24.087), 16.160–(95% CI: 6.354–41.097), and 24.714-fold (95% CI: 12.331–49.531) higher risks of having oral cancer compared to individuals with WT homozygotes who did not chew betel nut (Table 3).

**Table 3:** Adjusted odds ratio (AOR) and 95% confidence interval (CI) of oral cancer associated with *CD44* genotypic frequencies and betel nut chewing among 731 smokers.

Variable	Controls (n = 220) (%)	Patients (n = 511) (%)	OR (95% CI)	AOR (95% CI)
**rs1425802**				
aAA genotype & non-betel nut chewing	65 (29.5%)	30 (5.9%)	1.00	1.00
bAG or GG genotype or betel nut chewing	109 (49.5%)	191 (37.4%)	3.797 (2.320–6.212)	5.443 (2.919–10.148)
cAG or GG genotype with betel nut chewing	46 (20.9%)	290 (56.8%)	13.659 (8.017–23.272)	21.205 (10.901–41.248)
**rs187115**				
aAA genotype & non-betel nut chewing	107 (48.6%)	44 (8.6%)	1.00	1.00
bAG or GG genotype or betel nut chewing	90 (40.9%)	277 (54.2%)	7.485 (4.898–11.437)	10.519 (6.154–17.979)
cAG or GG genotype with betel nut chewing	23 (10.5%)	190 (37.2%)	20.089 (11.507–35.070)	37.631 (18.366–77.106)
**rs713330**				
aTT genotype & non-betel nut chewing	129 (58.6%)	67 (13.1%)	1.00	1.00
bTC or CC genotype or betel nut chewing	82 (37.3%)	376 (73.6%)	8.829 (6.039–12.906)	11.144 (6.923–17.937)
cTC or CC genotype with betel nut chewing	9 (4.1%)	68 (13.3%)	14.547 (6.836–30.959)	23.333 (9.461–57.544)
**rs11821102**				
aGG genotype & non-betel nut chewing	124 (56.4%)	69 (13.5%)	1.00	1.00
bGA or AA genotype or betel nut chewing	84 (38.2%)	394 (77.1%)	8.429 (5.782–12.289)	13.055 (7.984–21.348)
cGA or AA genotype with betel nut chewing	12 (5.5%)	48 (9.4%)	7.188 (3.578–14.443)	10.295 (4.400–24.087)
**rs10836347**				
aCC genotype & non-betel nut chewing	133 (60.5%)	68 (13.3%)	1.00	1.00
bCT or TT genotype or betel nut chewing	79 (35.9%)	388 (75.9%)	9.606 (6.574–14.038)	13.455 (8.292–21.832)
cCT or TT genotype with betel nut chewing	8 (3.6%)	55 (10.8%)	13.447 (6.059–29.840)	16.160 (6.354–41.097)
**rs13347**				
aCC genotype & non-betel nut chewing	73 (33.2%)	36 (7.0%)	1.00	1.00
bCT or TT genotype or betel nut chewing	116 (52.7%)	251 (49.1%)	4.388 (2.782–6.921)	5.703 (3.203–10.156)
cCT or TT genotype with betel nut chewing	31 (14.1%)	224 (43.8%)	14.652 (8.470–25.348)	24.714 (12.331–49.531)

The odds ratios (ORs) with their 95% confidence intervals were estimated by logistic regression models.

The adjusted odds ratios (AORs) with their 95% confidence intervals were estimated by multiple logistic regression models after controlling for age, gender and alcohol consumption.

a Individual with wild genotype but without betel nut chewing.

b Individual with either at least one mutated genotype or betel nut chewing.

c Individual with both at least one mutated genotype and betel nut chewing.

**Table 4:** Adjusted odds ratio (AOR) and 95% confidence interval (CI) of oral cancer associated with *CD44* genotypic frequencies and smokers among 552 betel nut consumers.

Variable	Controls (n = 93) (%)	Patients (n = 459) (%)	OR (95% CI)	AOR (95% CI)
**rs1425802**				
aAA genotype & non-smoker	6 (6.5%)	7 (1.5%)	1.00	1.00
bAG or GG genotype or smoker	41 (44.1%)	162 (35.3%)	3.387 (1.080–10.621)	5.454 (1.031–28.854)
cAG or GG genotype with smoking	46 (49.5%)	290 (63.2%)	5.404 (1.739–16.794)	8.004 (1.530–41.854)
**rs187115**				
aAA genotype & non-smoker	16 (17.2%)	16 (3.5%)	1.00	1.00
bAG or GG genotype or smoker	54 (58.1%)	253 (55.1%)	4.685 (2.207–9.945)	5.070 (1.880–13.671)
cAG or GG genotype with smoking	23 (24.7%)	190 (41.4%)	8.261 (3.649–18.699)	13.497 (4.554–40.002)
**rs713330**				
aTT genotype & non-smoker	17 (18.3%)	19 (4.1%)	1.00	1.00
bTC or CC genotype or smoker	67 (72.0%)	372 (81.0%)	4.968 (2.457–10.045)	9.313 (3.342–25.947)
cTC or CC genotype with smoking	9 (9.7%)	68 (14.8%)	6.760 (2.602–17.563)	16.111 (4.359–59.541)
**rs11821102**				
a GG genotype & non-smoker	19 (20.4%)	21 (4.6%)	1.00	1.00
bGA or AA genotype or smoker	62 (66.7%)	390 (85.0%)	5.691 (2.895–11.188)	7.609 (3.129–18.502)
cGA or AA genotype with smoking	12 (12.9%)	48 (10.5%)	3.619 (1.492–8.779)	4.228 (1.328–13.463)
**rs10836347**				
aCC genotype & non-smoker	16 (17.2%)	21 (4.6%)	1.00	1.00
bCT or TT genotype or smoker	69 (74.2%)	383 (83.4%)	4.229 (2.102–8.509)	5.077 (2.016–12.785)
cCT or TT genotype with smoking	8 (8.6%)	55 (12.0%)	5.238 (1.953–14.047)	4.899 (1.425–16.850)
**rs13347**				
aCC genotype & non-smoker	9 (9.7%)	11 (2.4%)	1.00	1.00
bCT or TT genotype or smoker	53 (57.0%)	224 (48.8%)	3.458 (1.364–8.768)	8.636 (2.289–32.587)
cCT or TT genotype with smoking	31 (33.3%)	224 (48.8%)	5.912 (2.269–15.404)	14.468 (3.778–55.411)

The odds ratios (ORs) with their 95% confidence intervals were estimated by logistic regression models.

The adjusted odds ratios (AORs) with their 95% confidence intervals were estimated by multiple logistic regression models after controlling for age, gender and alcohol consumption.

a Individual with wild genotype but without smoking.

b Individual with either at least one mutated genotype or smoking.

c Individual with both at least one mutated genotype and smoking.

Among betel-nut consumers in our cohort, subjects with CD44 polymorphic rs1425802, rs187115, rs713330, rs11821102, rs10836347 and rs13347 genes and who smoked had corresponding 8.004–(95% CI: 1.530–41.854), 13.497–(95% CI: 4.554–40.002), 16.111–(95% CI: 4.359–59.541), 4.228–(95% CI: 1.328–13.463), 4.899–(95% CI: 1.425–16.850), and 14.468-fold (95% CI: 3.778–55.411) higher risks of having oral cancer compared to betel-quid chewers with the WT gene who did not smoke (Table 4). Moreover, people who were either polymorphic for CD44 in 6 loci (rs1425802, rs187115, rs713330, rs11821102, rs10836347 and rs13347) or who smoked were at a 5.070–9.313-fold risk (*p*<0.05) of developing oral cancer, compared to people with the WT gene who did not smoke (Table 4). In light of the above results, we suggest that CD44 gene polymorphisms have strong impacts on oral-cancer susceptibility in betel-nut and/or smoking consumers.

To clarify the role of CD44 gene polymorphisms in oral cancer clinicopathologic statuses, such as tumor node metastasis clinical stage, tumor size, lymph node metastasis, distant metastasis, and cell differentiation, the distribution frequency of clinical statuses and CD44 genotype frequencies in oral cancer patients were estimated. No significant association between rs1425802, rs713330, rs11821102, rs1836347, and rs13347 gene polymorphisms and the clinicopathologic statuses was observed. However, among the 599 oral cancer patients, those with the polymorphic rs187115 gene had a higher risk of developing stage III or IV OSCC (adjusted odds ratio (OR) = 1.619; 95% CI = 1.124–2.333) compared with the patients with the rs187115 WT, but no difference in tumor size, lymph node metastasis, distant metastasis, or cell differentiation was observed (Table 5).

**Table 5:** Distribution frequency of clinical status and *CD44* rs187115 genotype frequencies in 599 patients with oral cancers.

	genotypic frequencies			
Variable	AA(N = 336) n (%)	AG+ GG(N = 263) n (%)	OR (95% CI)	AOR (95% CI)
Clinical Stage				
Stage I/II	163 (48.5%)	101 (38.4%)	1.00	1.00
Stage III/IV	173 (51.5%)	162 (61.6%)	**1.511 (1.089–2.098) ** ***p*** ** = 0.013** *	**1.619 (1.124–2.333) ** ***p*** ** = 0.010** *
Tumor size				
≦ T2	212 (63.1%)	151 (57.4%)	1.00	1.00
> T2	124 (36.9%)	112 (42.6%)	1.268 (0.912–1.764) *p* = 0.158	1.314 (0.910–1.899) *p* = 0.145
Lymph node metastasis				
No	217 (64.6%)	165 (62.7%)	1.00	1.00
Yes	119 (35.4%)	98 (37.3%)	1.083 (0.774–1.515) *p* = 0.641	1.075 (0.742–1.557) *p* = 0.702
Distant metastasis				
No	334 (99.4%)	257 (97.7%)	1.00	1.00
Yes	2 (0.6%)	6 (2.3%)	3.899 (0.780–19.477) *p* = 0.074	9.427 (0.892–99.624) *p* = 0.062
Cell differentiation				
Well	42 (12.5%)	101 (14.4%)	1.00	1.00
Moderately or poorly	294 (87.5%)	162 (85.6%)	0.846 (0.528–1.356) *p* = 0.487	0.905 (0.543–1.506) *p* = 0.700

The ORs with 95% CIs were estimated by logistic regression models.

The AORs with 95% CIs were estimated by multiple logistic regression models after controlling for age, gender, alcohol consumption, and tobacco use.

> T2: tumor size >2 cm in the greatest dimension.

*p value <0.05 as statistically significant.

## Discussion

In this study, we provided novel information on the effects of CD44 on oral cancer susceptibility, and elucidated the interactions of environmental risk factors and clinicopathologic statuses.

Alcohol consumption, betel-quid chewing, and tobacco smoking are the primary environmental risk factors for oral cancer. In the present study, higher ratios of individuals who had chewed betel quid, consumed alcohol and smoked tobacco, were observed in the group of OSCC patients (76.6%, 59.4%, and 85.3%, respectively) compared with the controls (16.6%, 38.1%, and 39.2%, respectively). Thus, betel-quid chewing, alcohol consumption and tobacco smoking are indicated to be highly correlated with an increased risk of oral cancer. Moreover, Lu et al reports that more male group chewed areca nut than female group (9.2% vs 0.9%) in Taiwan studies, which should be a major contributor to the higher oral cancer incidence of males since areca nut chewing is a well-recognized risk factor for oral cancer [28].

In oral cancer, CD44 expression has been observed to have a significant association with heavy smoking and/or alcohol consumption, thereby predicting poor prognoses. A statistically significant association between smoking and CD44 expression has been observed in SCCs located in the orolarynx, hypopharynx, and larynx [29]. Areca quid has been considered a carcinogen to humans, and it is an independent risk factor in OSCC development among people who chew it in certain regions of Asia. The betel quid used in Taiwan contains areca nut, lime, and piper betel inflorescence or leaf [30]. However, a study by Kuo et al indicated that loss of CD44v7-8 expression might be a valuable factor for determining the prognosis in OSCC patients, whereas no significant correlation was observed between CD44v7-8 expression and the daily or total consumption of betel quids or smoking of cigarettes by OSCC patients [31].

The expression of CD44 and its relationship with tumor prognosis remains controversial. Certain studies have indicated that the overexpression of CD44 in tumors is correlated with increased resistance to radiation therapy and an elevated risk of local recurrence [32]–[34], whereas others have linked poor prognosis to CD44 downregulation on tumor cells, particularly in OSCC [35], [36]. Recent studies have associated increased CD44 expression with higher OSCC aggressiveness [15], [16], [37], [38]. However, thus far, reports on CD44 SNP expression in OSCC have been limited. In this study, we sought to determine the genetic variants of CD44 that may confer a risk of OSCC in 599 patients and 561 healthy controls. The data in Table 2 show that those with the CD44 polymorphism rs187115 variant genotypes (AG+GG) had a higher risk for OSCC compared with those with the WT genotype. Although the functional importance of CD44 SNP rs187115 has not been tested experimentally, an association with the risk of oral cancer is proposed based on the locations of the analyzed variants. However, in certain genes, an SNP arising in the coding, promoter, or regulatory region may have functional consequences [39].

CD44 SNP rs187115 is located in the first intron of CD44. Although no regulatory role for intron 1 of CD44 has been proposed, Zhou et al reported a similar intron 1 CD44 SNP in breast cancer and indicated that it may play a role in altered splicing of CD44 and effected expression level [22]. Furthermore, a previous study determined that CD44 SNP rs187115 possesses the greatest allelic difference in cellular growth response to standard chemotherapeutic agents, regardless of p53 mutational statues in all 59 tested cell lines [20]. In our study, the data revealed that rs187115 is associated with higher risk of stage III/IV but not with tumor size, metastasis of distant organ and/or lymph nodes, as shown in Table 5. However, AJCC classification illustrates the association between stages III/IV with larger tumor, distant metastasis and lymph node involvement. Such inconsistence could be resulted from that only 8 subjects with distant metastasis, which may profoundly affect the statistical analysis. Furthermore, the distribution of cases with lymph node involvement (N0) between stage II (T2N0M0) stage III (T3N0M0) may also contribute to the abovementioned inconsistence.

Several studies have implicated that CSCs play a central role in cancer progression, invasion, and disease recurrence after therapy, and the cause of high morbidity and, ultimately, death in most patients with oral cancer is distant metastasis [40], [41]. CSCs in OSCCs have been observed and defined using multiple methodologies, such as cell lines, patient-derived xenografts, and primary tumor specimens [42]. CD44 is a CSC gene and has been observed to be highly expressed in the poorly differentiated metastatic stages of oral cancer patients, leading to invasion, malignancy, and poor prognosis [43]. Furthermore, oral CSCs have been considered to have inherent drug and radiation resistance that counteracts the effect of conventional therapies, which leads to tumor recurrence [42], [44]. A previous study demonstrated that the CD44^high^ subpopulation from oral cancer cells represents increased viability and survival compared with CD44^low^ cells after treatment with a panel of cytotoxic drugs, including 5-FU, cisplatin, docetaxel, paclitaxel, and carboplatin, as well as radiation [45]. In our study, because we observed the significant association of CD44 SNP rs187115 functional variant G with oral cancer susceptibility in stage III and IV OSCC patients, and CD44 SNP rs187115 was observed to be associated with chemoresistance [20], we proposed that the oral CSC characteristics of CD44 contributed to the chemoresistance of the rs187115 polymorphism in oral cancer, and the CD44 SNP rs187115 polymorphism might act as a marker to predict poor prognosis in OSCC patients. We also observed that the expression of the CD44 rs13347 polymorphism in breast cancer patients in a previous study [23] differed from that in OSCC patients in our study. It has been proposed that it is not the mutation of CD44, but the factors promoting carcinogenesis, that control the patterns of the misregulated CD44 in most cancers [46]. These factors include mitogenic signals, such as the Ras-MAP cascade, which controls the alternative splicing of CD44, [47], [48] and the loss of various subunits of the SWI/SNF chromatin remodeling complex that leads to the loss of CD44 transcription [49], [50]. Although the exact mechanism is unclear, it is possible that even the CD44 expressed in tumors such as breast and oral cancer exhibit the same CSC characteristics, because various detailed mechanisms and signaling pathways are involved in CD44 regulation. However, well-designed studies are required to determine the role of the signaling pathway of CD44 regulation in tumor aggressiveness and CSCs.

One of the limitations to our study is that we categorized patients as “ever-users” and “never-users” for the information on certain environmental risk factors, such as alcohol use, betel nut chewing, and tobacco smoking. Therefore, the amount, length, and past history of betel nut, tobacco, and alcohol use were not available and a detailed analysis could not be performed. In addition, our data collection relied on self- reports, and patients might conceal or be reluctant to report their actual use of such substances. Thus, residual confounding effects might have been caused by the misclassification of alcohol, betel nut, and tobacco use. Other information, such as medicinal nicotine use and familial and heredity risks were also not available, and these limitations might have restricted the adjustment for possible confounding factors. Increasing the specimen number and accounting for more OSCC risk factors in the analysis might accurately validate these findings in a future study.

In conclusion, our results suggested that gene–environment interactions between the CD44 polymorphism and betel quid chewing and tobacco smoking alter the susceptibility to oral cancer development. Based on a review of the relevant literature, our study is the first to demonstrate a significant association between the CD44 rs187115 A/G polymorphism and the risk of oral cancer. Patients who carried the functional CD44 variant rs187115 G might possess greater chemoresistance to advanced- to late-stage oral cancer than WT carriers do, and CD44 rs187115 might act as a marker to predict poor prognoses in OSCC patients.
